# Baseline verbal fluency performance as predictor of state anxiety during a live hand-grenade throwing exercise – A prospective study of Swedish military conscripts

**DOI:** 10.1186/1744-9081-3-39

**Published:** 2007-08-13

**Authors:** Magnus R Larsson, Per-Olof Michel, Martin Bäckström, Aki Johanson

**Affiliations:** 1 Department of Psychology, Lund University, Box 213, SE-221 00 Lund, Sweden; 2 Järnvägsgatan 8, SE-652 25 Karlstad, Sweden

## Abstract

**Background:**

We investigated whether individual differences in baseline executive control capacity could predict state anxiety during a potentially life-threatening situation.

**Methods:**

19 Swedish military conscripts were assessed during two measurement occasions. During a baseline measurement, data regarding performance on a letter fluency task and state anxiety were assessed. During a second measurement, performed immediately prior to participation in a live hand-grenade throwing exercise, data regarding state anxiety was assessed. All participants were male, right-handed and had fulfilled 12 years of education.

**Results:**

The level of state anxiety was significantly increased between the two measurement occasions (*p *< .01). Both the number of words produced (*β *= -.37; *p *< .05) and the number of perseveration made (*β *= .43; *p *< .05) on the verbal fluency task predicted, while controlling for state anxiety at baseline, the level of experienced state anxiety during the threatening situation.

**Conclusion:**

Although more research is needed the present finding suggests that individual differences in executive control capacity might be related to emotion regulation ability during acute stressor exposure.

## Background

The concept of executive control refers to the top-down control of cognitive processes in which internal states and intentions are used to guide behaviour [1]. One very common conceptualization of executive control is Baddeley's working memory model in which the central executive component is characterized as an attentional control system [2]. Another common conceptualization has been presented by Cohen and Miller [1] who defines executive control as the cognitive mechanism that coordinates sensory and motor processes in response to an internal goal. One of few theories of executive control that differentiate the concept on a conceptual and a methodological level is Shimamura's [3] dynamic filtering theory. In this theory the concept of executive control is defined as the monitoring, selection and control of cognitive processes. Shimamura identifies four domains of executive control. The first domain, selection, is referring to the ability to direct attention towards a perceptual stimulus or a representation in memory. The second domain, maintenance, is referring to the ability to hold selected information active. Further, the third domain, updating, is referring to the modulation and reorganization of information in working memory. Finally, the domain of rerouting is referring to the capacity to shift attention between different response sets.

One cognitive function that is dependent upon the domains of executive control is the retrieval of semantic knowledge [4]. In neuropsychological testing this is usually assessed with some form of verbal fluency tasks. Two common types of verbal fluency tasks are letter fluency and semantic fluency [5]. In the former the participants' task is to generate words beginning with a specific letter and in the latter the participants' task is to generate words belonging to a specific category. Empirical evidence has been presented suggesting that both letter and semantic fluency tasks are dependent upon semantic facilitation [6] and executive processes [6,7]. As pointed out by Shimamura [4] verbal fluency tasks requires the ability to selectively focus attention on a semantic category, the capacity to "on-line monitor" previously recalled words and also to continuously update the words that have been used. Similar descriptions has also been presented by Rende, Ramsberger and Miyake [7] who states that verbal fluency tests require an adequate mental set shifting ability which guides the strategic search of words and Elfgren and Risberg [8] who states that a verbal fluency task requires an ability to internally generate responses and, at the same time, plan and follow rules. Performance on verbal fluency tasks [5] is commonly assessed by the total amount of words produced during a specific time period and/or the relative frequency of error types. One such error type is recurrent perseveration which is characterized by prior responses being repeated during the task. No study, known to the authors, has so far investigated whether individual differences in the ability to retrieve semantic knowledge during a non-stress or baseline situation can be used to predict level of subjective state anxiety during exposure to an acute stressor. State anxiety is defined by Spielberger, Gorsuch, Lushene, Vagg, and Jacobs [9] as a temporal emotional reaction varying in intensity with the subjective experience of threat during the situation. The reaction is characterized by worry, nervousness and tension and an increase in arousal due to the activation of the autonomic nervous system.

The question whether individual differences in the ability to retrieve semantic knowledge during non-stress can be used to predict level of subjective state anxiety during acute stressor exposure is relevant for two reasons. First, it has been suggested that state anxiety influences cognitive capacity [10] but very few studies have investigated the possible impact of baseline executive control capacity on state anxiety during acute stressor exposure. Due to its dependence upon executive control it seems reasonable to assume that the ability to retrieve semantic knowledge, as measured by verbal fluency, can be used to operationalize individual differences in executive control. In this context, studies investigating Baddeley's [2] working memory model have shown empirical evidence indicating that individual differences in the ability to control attention influence other cognitive functioning. For example, in a study of semantic verbal fluency Rosen and Engle [11] showed that individuals with high-span working memory generated an increased amount of words in comparison to individuals with low-span working memory. This finding was interpreted in terms of individuals with high-span working memory having enough capacity to both generate new words and self-monitor for repetitions. Further, in a study by Brewin and Smart [12] it was showed that individuals with high working memory capacity had fewer intrusions during a thought suppression task than individuals with low working memory capacity. Based on the findings by Rosen and Engle [11] and Brewin and Smart [12] it seems reasonable to assume that individual differences in baseline executive control might be related to available cognitive resources to deal with exposure to an acute stressor. Despite a lack of direct empirical evidence in support of this hypothesis, it has gained some indirect empirical support from clinical stress research investigating symptoms of posttraumatic stress disorder. For example, in a study by Parslow and Jorm [13] results were presented showing that a higher pre-trauma performance on measurements of word digit span, vocabulary, coding speed and word recall three years before exposure to a natural disaster were related to lower post-trauma symptoms of reexperiencing and hyperarousal. Second, if individual differences in executive control can be used to prospectively predict state anxiety during real-life highly stressful situations then cognitive testing may provide more fine-grained methods to predict stress reactions in, for example, the recruiting process to high-stress occupations such as the military- or police force.

In the current study a prospective design is used to investigate whether individual differences in baseline executive control can be used to predict subjective state anxiety during acute stressor exposure in a sample of Swedish military conscripts. Individual differences in executive control were operationalized by performance on a letter fluency task. The stressful situation investigated is a live hand-grenade throwing exercise. This type of exercise is one of the most dangerous and stressful during the soldiers' basic training and can, if a mistake is made, lead to serious injury or death. It was hypothesized that both a low performance and amount of perseverations made on the letter fluency task should, when controlling for state anxiety during baseline, predict an increased level of state anxiety during the hand-grenade throwing exercise.

## Methods

### Participants

19 soldiers (age *M *= 18.53; *SD *= 0.51) participated in the study. All participants were male and did their compulsory military service at the seventh armoured regiment (P 7) localized outside Lund in the southern part of Sweden. The participants had served approximately one month before participating in the study and had all been selected to serve as squad-leaders during the previous general selection to Swedish military service. All participants had before entering the military system fulfilled 12 years of education. No participant yet had enrolled into any kind of higher studies. Further, all participants were right handed. Four additional participants were tested in the study but were excluded in the analysis. The first participant was excluded due to an accident that had damaged the brain areas involved in taste and smell functions. The second participant was excluded due to disturbance during the verbal fluency testing. The third and fourth participants were excluded due to being both left and right handed.

### Material

#### State anxiety

State anxiety was measured by Spielberger Trait State Anxiety Inventory (STAI-S) [9]. STAI-S contains 20 statements all intended to measure level of state anxiety during a specific situation. The participant's task is to estimate the statements on a four point response scale ranging from the alternative "Not at all" to "Very much". Cronbach's alpha was .90 for the baseline measure and .90 for the stressful situation indicating good consistency of the scale during both measurements.

#### Controlled Oral Word Association Test

Verbal fluency was assessed by the Controlled Oral Word Association Test (COWAT) [14]. In this test the participants are instructed to, during one minute each, generate words beginning with the three different letters F, A and S. All words, except proper nouns (which, in our case, also included names on cities, names on countries and digits), are allowed. However, no correction is made during the testing session if this rule is violated. In the current study, the total number of words generated over the three letters was used in the analysis. Besides that, the number of pair-wise perseverations of same words was used in the analysis (i.e. if the participants, for example, stated the word "sun" twice then this constituted one perseveration).

### Procedure

A first baseline measurement session was performed at the regiment during the week before the live grenade throwing exercise. During this session, all participants were tested individually. Primarily, the participants were interviewed regarding their level of education, handedness, possible neurological diseases or psychiatric disorders and medication. This was performed as a structured interview. Following this, the participants were tested on the COWAT and thereafter the STAI-S was administered. Regarding the latter, the participants were instructed to fill in the questionnaire at their barrack directly after the testing and return it to the researcher (MRL) as quick as possible. During a second measurement session, conducted a few minutes before the onset of the live hand-grenade throwing exercise, the participants were again administered the STAI-S by one of the authors (MRL) in a bunker localized approximately 10 meters from the grenade throwing area. All participants completed the questionnaire in the bunker before the throwing session began. Thereafter each participant, one at the time, approached the grenade throwing area in which the commanding officer of the exercise waited. In the throwing area the commanding officer instructed each participant about the throwing procedure and what to do if the throwing failed or the grenade did not explode. After that one practice grenade was thrown and this was followed by the throwing of two live grenades.

#### Statistical procedure

Bivariate correlations were used to investigate the relationship between the total number of words produced and the number of perseverations made during the letter fluency task. Two standard bivariate regression analyses were performed to investigate the relationship between state anxiety during the baseline measurement and the verbal fluency measures. In these analyses state anxiety during baseline was used as the independent variable while the number or words produced and the number of perseverations made were used as dependent variables. A t-test for dependent means was used to compare state anxiety during the baseline measurement and the grenade throwing exercise. Finally, a hierarchical multiple regression analysis was performed to investigate the predictive power of baseline state anxiety and baseline verbal fluency on state anxiety during the stressful situation. In this analysis the independent variables were entered into two blocks. In the first block state anxiety during baseline was entered. In the second block the number of words produced and the number of perseverations made were entered into the equation. According to the assumptions for multiple regression presented by Tabachnick and Fidell [15] the occurrence of uni- and multivariate outliers, the normal distribution of the residuals and the occurrence of multicollinearity and singularity were investigated. All scores above and below three standard deviations from the mean were considered as univarite outliers and rescored to the first integer under or over the three standard deviation limit. Further, to detect the occurrence of multivariate outliers Mahalanobis distance (χ^2 ^= .001) was calculated for every participant. The maximum value was 7.10 indicating distance to the critical value of 16.27. The visual inspection of the residuals showed a normal plot indicating no major deviation from normal distribution. Multicollinearity was detected by calculating tolerance levels for all variables (1-SMC). The lowest value obtained was .88 implicating no problem with multicollinearity in any variable. Furthermore no singularity among the variables was detected. Finally, to safeguard for skewness all variables in the multiple regression analysis were transformed to rank-ordered variables and tested into the regression analysis. No difference regarding significance/non-significance was found.

## Results

### Descriptive statistics of verbal fluency performance

In Table 1 descriptive statistics of the verbal fluency measures are presented. As can be seen in the table, the highest and lowest number of words produced was 64 and 28, respectively. Further, the highest number of perseverations made was 5. No significant bivariate relationship was found between the number of words produced and the number of perseverations made (*r *= .21; *p *> 0.05).

**Table 1:** Descriptive statistics of verbal fluency performance

Variable	*M*	*SD*	*Min.*	*Max.*
Number of words	45.00	11.54	28	64
Perseverations	1.21	1.62	0	5

### Descriptive statistics of state anxiety

The level of state anxiety reported during the baseline testing session was *M *= 34.29 (*SD *= 10.58). The level of state anxiety reported prior to the hand-grenade exercise was *M *= 40.24 (*SD *= 9.09). The highest raw-score reported during the hand-grenade exercise was 58. The level between the two measures of state anxiety was significantly increased (*t *(18) = -3.06; *p *< .01). Further, the bivariate relationship between the two measures was *r *= .66 (*p *< .01). Finally, the result from the standard regression analyses revealed that state anxiety during baseline did not predict neither the number of words produced (*β *= -.14; *p *> .05)nor the number of perseverations made (*β *= .25; *p *> .05).

### Prediction of state anxiety during the live hand-grenade throwing exercise

In Table 2 the result of the multiple regression analysis predicting state anxiety during the hand-grenade throwing exercise is presented. As can bee seen in the table the entering of both blocks provided a significant contribution to the model. *R*^2 ^for the first block was .43 and for the second block .66 indicating a 23 percent increase in explained variance when the verbal fluency measures were entered into the equation. This change was also statistically significant. (*F *change (2.13) = 4.54; *p *< .05). As can be seen in the second block, state anxiety during the baseline measurement, the total number of words produced and the number of perseverations made during the verbal fluency task significantly predicted the level of state anxiety during the hand-grenade throwing exercise. Baseline state anxiety was positively related to state anxiety during the second measurement indicating that participants with high state anxiety during the baseline measurement also had a higher state anxiety during the grenade exercise. The number of words produced was negatively related to state anxiety during the second measurement suggesting that participants with a higher verbal fluency during baseline experienced less subjective state anxiety during the stressful situation. Finally, the number of perseverations made was positively related to state anxiety indicating that participants with a higher amount of perseverations during baseline experienced more state anxiety during the stressful situation.

**Table 2:** Summary of multiple regression analysis predicting state anxiety during hand-grenade throwing

Variable	*B*	*SE B*	*β*	*p*
Baseline state anxiety	.56	.17	.66	.004

Baseline state anxiety	.42	.14	.50	.012
Number of words	-.31	.14	-.37	.043
Perseverations	2.27	.91	.43	.027

Note: *R*^2 ^= .43 for Step 1 (*F *= 11.26; *p *< .01); *R*^2 ^= .66 for Step 2 (*F *= 8.56; *p *< .01)

## Discussion

The current study investigated whether baseline performance on a letter fluency task could be prospectively used to predict subjective state anxiety during a potentially life-threatening situation. It was hypothesized that if the capacity for executive control is related to the degree of state anxiety experienced during a highly stressful situation then a low baseline performance and/or a high number of perseverations should predict an increased level of state anxiety during a live hand-grenade throwing exercise. The hierarchical regression analysis supported the general hypothesis that capacity for executive control was related to level of state anxiety. More specifically and in accordance with the hypothesis the result showed that the total number of words produced during the baseline measurement predicted level of subjective state anxiety indicating that a higher capacity to focus attention on a semantic category is related to a lower level of state anxiety. Further, a high number of recurrent perseverations predicted a higher level of state anxiety indicating that a low ability to maintain and update previous responses was associated with a more pronounced anxiety reaction. It was also found that performance on the verbal fluency task was not significantly predicted by state anxiety during the baseline measurement session implicating no effect of state anxiety on the verbal fluency performance. Importantly, state anxiety during baseline was also controlled for in the analysis suggesting no effect of this variable on the relationship between baseline verbal fluency performance and state anxiety during acute stressor exposure.

Overall, the results support the suggestion that individual differences in executive control capacity is related to the experience of state anxiety during acute stressor exposure and that this relationship is independent of the level of state anxiety experienced during the baseline measurement. The results can be interpreted to support a theoretical model of executive control capacity mediating the emotional experience of state anxiety during acute stressor exposure. Clearly, the results should, however, be replicated with an experimental design before further conclusions about the possible influence of executive control on state anxiety can be drawn.

Assuming that the number of words produced is mirroring individual differences in the ability to direct attention towards a perceptual stimulus or a representation in memory then it might be that this executive function may be used to regulate emotion. Studies using different neuroimaging techniques [16-18] have shown that performing on letter fluency tasks activates regions in the left prefrontal cortex. It has also been implicated [19] that the left hemisphere is involved in the control of emotion and in the modulation of impulsive emotional expression. Thus an effective capacity to direct attention towards perceptual stimuli or memory representations may be related to a better retrieval or use of verbal coping strategies which increase the possibility to experience control in potentially stressful and threatening situations. As shown by Ochsner et al. [20] up- and down regulation of emotion modulate the activity in the left hemisphere amygdala which might mirror the use of verbal strategies. In line with this reasoning it has been suggested [21] that a high working memory capacity is related to an increased ability to resist attending to negative information. An ability to shift attention from a threatening to a non-threatening cue is relevant to emotion regulation and suggests that individuals with a high working capacity are characterized by a more effective attentional control. Interestingly, results have been presented [22] showing that individual differences seem to exist between individuals with a high degree of trait anxiety and high attentional control and individuals with a high degree of trait anxiety and low attentional control regarding attentional disengagement from threatening stimuli.

Further, the finding that an increased number of recurrent perseverations predicted a higher state anxiety supports the suggestion that individuals with a low ability to maintain and update previous responses experience more state anxiety during a stressful situation. However, the non-significant relationship between the total amount of words produced and the amount of recurrent perseverations indicate that these types of performances are dependent upon different executive control domains. As such this finding supports the theoretical model by Shimamura [3]. In accordance with the emotion regulation hypothesis discussed above it also seems reasonable to assume that a difficulty to maintain and update active responses may interfere with the emotion regulation process. Speculatively, a difficulty in the inhibition of active responses might be related to increased problems to suppress automatic negative thoughts during stressful situations. Such problems can be expected to interfere with the ability to use executive control in the coping process and to induce an increased state anxiety reaction.

The results of the current study suggest that letter fluency tasks might be used to recruit individuals whom are resistant to experience high state anxiety during acute stressor exposure. Thus, the results could be useful when recruits to military combat units, peacekeeping service or the police force are selected. However, as pointed out above, more research is needed to investigate the relationship between executive control and the experience of anxiety during acute stressor exposure before any further conclusions can be made.

### Limitations

Finally, the current study suffers from some limitations. First, it is not possible to exclude the possibility that a negative mood might have influenced performance on the verbal fluency task. It has, for example, been shown [23] that participants in a dysphoric mood perform worse on a letter fluency task than on a figure fluency task implicating a decreased left frontal lobe functioning when experiencing negative affect. Since the soldiers participating in the study only had served a month it is possible that the changeover from civilian to military life has had a negative effect on their mood. However, it seems reasonable to assume that the non-significant predictions between baseline state anxiety and the letter fluency performance indicates a minor impact of mood on the neuropsychological testing during the non-stress situation. Second, although letter fluency could be considered a task requiring executive control it might be that other tests more adequately capture the dimensions of executive control. Third, some empirical evidence exists supporting the view that state anxiety is a multidimensional construct (see for example Endler and Kocovski, [24] or Spielberger, [25]). Since we did not operationalize state anxiety as a multifaceted construct it is possible that the grenade throwing exercise does not pinpoint a subjective experience of life-threat but instead mirrors a subjective experience of social threat. Fourth, since we only measured level of subjective state anxiety and not performance in the throwing situation then important data might have been missed. In an applied perspective it seems reasonable to assume that a faulty performance in the throwing situation, due to stressor induced state anxiety, may have more serious consequences than the subjective experience of stress. Fifth, no females participated in the study making conclusions about possible gender differences impossible. Sixth, the low number of participants may implicate a problem of statistical power. However, an argument against this threat is that a low statistical power increases the risk of making a type II error (i.e. the null hypothesis is obtained although it should be rejected [15]) and as such it could be expected that no significant effects should be found. Since all predictions were significant it could therefore be assumed that the results implicate relatively strong relationships.

## Conclusion

The current study suggests that individual differences in letter fluency during non-stress conditions can predict the experience of state anxiety during acute stressor exposure. As such the results may support a relationship between baseline executive control capacity and emotion regulation ability. However, the results should be replicated before further conclusions about the relationship between individual differences in executive control and the experience of state anxiety can be drawn.

## Competing interests

The author(s) declare that they have no competing interests.

## Authors' contributions

MRL was the primary investigator and the author of this study. POM contributed with valuable comments on the content of the manuscript. MB participated in the statistical analyses of the results and provided valuable comments on the content of the manuscript. AJ participated in the development of the design and provided valuable comments on the content of the manuscript.
